# bHLH–PAS Proteins: Their Structure and Intrinsic Disorder

**DOI:** 10.3390/ijms20153653

**Published:** 2019-07-26

**Authors:** Marta Kolonko, Beata Greb-Markiewicz

**Affiliations:** Department of Biochemistry, Faculty of Chemistry, Wroclaw University of Science and Technology, Wybrzeże Wyspiańskiego 27, 50-370 Wroclaw, Poland

**Keywords:** bHLH–PAS transcription factor, intrinsically disordered region, IDR, C-terminus

## Abstract

The basic helix–loop–helix/Per-ARNT-SIM (bHLH–PAS) proteins are a class of transcriptional regulators, commonly occurring in living organisms and highly conserved among vertebrates and invertebrates. These proteins exhibit a relatively well-conserved domain structure: the bHLH domain located at the N-terminus, followed by PAS-A and PAS-B domains. In contrast, their C-terminal fragments present significant variability in their primary structure and are unique for individual proteins. C-termini were shown to be responsible for the specific modulation of protein action. In this review, we present the current state of knowledge, based on NMR and X-ray analysis, concerning the structural properties of bHLH–PAS proteins. It is worth noting that all determined structures comprise only selected domains (bHLH and/or PAS). At the same time, substantial parts of proteins, comprising their long C-termini, have not been structurally characterized to date. Interestingly, these regions appear to be intrinsically disordered (IDRs) and are still a challenge to research. We aim to emphasize the significance of IDRs for the flexibility and function of bHLH–PAS proteins. Finally, we propose modern NMR methods for the structural characterization of the IDRs of bHLH–PAS proteins.

## 1. Introduction to bHLH–PAS Proteins

The basic helix–loop–helix/Per-ARNT-SIM (bHLH–PAS) proteins are a class of transcriptional regulators that commonly occur in living organisms. They play an important role in the regulation of a variety of developmental and physiological events [1]. The maintenance of cellular and systemic oxygen homeostasis is performed by hypoxia-inducible factor 1α (HIF1-α) [2]. In the hypoxia condition, HIF1-α is translocated to the nucleus [3] where it regulates transcription activity related to angiogenesis, cell proliferation/survival, glucose metabolism, and iron metabolism. The incorrect control of the listed processes is fundamental in many diseases, including cancer, strokes, and heart disease [2]. Some bHLH–PAS family members act as receptors for different high and low molecular ligands [1]. The only known small ligand-activated bHLH–PAS protein, aryl hydrocarbon receptor (AHR), is involved in toxin metabolism and binds highly toxic ligands, such as TCDD [4]. The ligated AHR migrates to the nucleus and mediates a wide range of biological responses to poisons. This mediation comprises a wasting syndrome, hepatotoxicity, teratogenesis, and tumor promotion [4]. Overexpression and constitutive activation of the AHR have been observed in various types of tumors [5]. Importantly, the AHR has been described as a critical modulator of host–environment interactions, especially for immune and inflammatory responses [6].

Another interesting example of a bHLH–PAS family member is the single-minded protein (SIM), which plays a significant role during central nerve cord [7] and genital imaginal disc development [8]. As shown, SIM gene mutations contribute to certain dysmorphic features of brain development and also the mental retardation in Down syndrome [9]. Interestingly, SIM overexpression is also associated with breast and prostate cancer [10], which indicates connections between their apparently unrelated signaling pathways.

Members of the bHLH–PAS family were shown to be targets for disease therapy. AHR, highly expressed in multiple organs and tissues, may influence tumorigenesis both by direct effect on the cancer cells and by modulation of the immune system. For this reason, the development of selective AHR modulators active against multiple tumors is a desirable direction of research [11]. Also, targeting of the HIF1-α pathway as a novel cancer therapy is a current project [12]. As AHR was shown to modulate the immune response in the respiratory tract, this protein can be potentially used also as a therapeutic object for the treatment of various inflammatory lung diseases [13,14]. Another member of the family, expressed mainly in the brain, neuronal PAS domain-containing protein 4 (NPAS4) has been proposed as a novel therapeutic target for depression and neurodegenerative diseases [15] and as a component of new stroke therapies [16]. Additionally, NPAS4, whose expression was also detected in the pancreas, was proposed to be a therapeutic target for diabetes [17] and as a treatment during pancreas transplantation [18].

**Figure 1:**
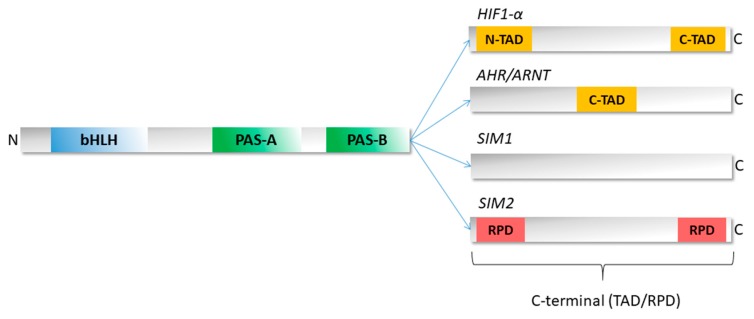
Schematic representation of the bHLH–PAS protein domain structure. The N-terminal part of bHLH–PAS proteins is characterized by the presence of defined domains: bHLH (blue), PAS-A and PAS-B (green). The C-terminal part presents significant diversity and contains variable transactivation/repression domains (TAD/RPD). The C-termini of selected proteins (HIF1-α, AHR/ARNT, SIM1, and SIM2) are presented. Yellow boxes indicate TADs while the red box indicates RPD. Based on [26,27,28,29].

In spite of performing a high diversity of functions, the bHLH–PAS proteins family exhibits a relatively well-conserved domain structure in the N-terminal part of their sequence (Figure 1). The bHLH region contains approximately 60 amino acid (aa) residues and can be divided into two functionally distinctive parts: the basic region responsible for DNA binding (approximately 15 aa), and the neighboring C-terminal HLH region, which takes part in protein dimerization [19]. The PAS domain is located in the central part of the protein and usually comprises about 300 aa residues [1]. It is divided into two structurally conserved regions named PAS-A and PAS-B, which are often connected to a single PAS-associated C-terminal (PAC) motif [20]. The PAS-A and PAS-B regions are separated by a poorly conserved link [1]. The PAS-A region is critical for selecting a dimerization partner and ensuring the specificity of target gene activation [21]. The PAS-B region is usually responsible for sensing diverse exogenous and endogenous signals, and is accompanied by energetic and conformational changes that regulate protein activity [21]. Contrary to conserved domains, the C-termini of bHLH–PAS proteins present significant variability [21] and contain variable transcription activation/repression domains (TAD/RPD) (Figure 1) [22,23]. An example is the mammalian SIM existing in two isoforms: SIM1 and SIM2. Both isoforms present a high amino acid identity in their N-termini (90% identity in the bHLH and PAS regions) and extreme diversity in their C-termini [24]. While SIM1 activates the expression of target genes, SIM2 acts as an inhibitor. Interestingly, the opposite transcriptional effect disappears after the deletion of both SIM1 and SIM2 C-termini, resulting in proteins with a similar activity [25,26]. Moffet and Pelletier [26] demonstrated that a distinct SIM2 C-terminal sequence comprises two repression domains with a high proline/serine and proline/alanine content, respectively. It is a feature of “repressor motifs”, which can also be found in a large number of other transcriptional repressors [25,26]. Due to the highly variable amino acid sequence and the lack of predefined domains, C-termini are believed to be responsible for the specific modulation of the functioning of bHLH–PAS proteins and the recognition of partner proteins necessary for their unique action [21].

Generally, bHLH–PAS proteins can be divided into two classes. While the expression of class I proteins is specifically regulated by diverse physiological states and/or environmental signals [30], class II proteins are expressed continuously and serve as heterodimerization partners for class I members. Only the dimer of the two bHLH–PAS proteins acts as a functional transcription factor complex, regulating the expression of genes under its control [22]. Mammalian bHLH–PAS transcription factors are listed in Table 1.

## 2. bHLH–PAS Protein Conservation between Organisms

bHLH–PAS proteins are highly conserved among different organisms, including vertebrates and invertebrates [33]. Most mammalian representatives possess orthologs in insect species. An example is the *Drosophila melanogaster* TANGO (TGO) protein, which is a homologue of the mammalian class II protein, ARNT [34]. TGO is known as the general dimerization partner for Similar (SIMA), Trachealess (TRH), Single-minded (SIM) protein, Spineless (SS), and Dysfusion (DYS), performing functions equivalent to mammalian ones. 

In 2017, the Nobel Prize in Physiology or Medicine was awarded to J. C. Hall, M. Rosbash, and M. W. Young for their discoveries of molecular mechanisms controlling the circadian rhythm in *D. melanogaster*. As shown, the two bHLH–PAS transcription factors CLOCK and CYCLE play a key role as transcriptional activators for *period* (*per*) and *timeless* (*tim*) genes [35,36]. Thanks to the conservation of circadian bHLH–PAS proteins between *D. melanogaster* and mammals [35], the explanation of the fly daily rhythm enabled the understanding of a similar, though much more complicated, process in mammals, controlled by two orthologous to CLOCK/CYCLE heterodimers: CLOCK/BMAL1 and NPAS-2/BMAL1 [22].

In spite of significant similarities, some exceptions between vertebrates and invertebrates can be noticed. The bHLH–PAS transcription factor, Methoprene-tolerant protein (MET), occurs exclusively in insects and to date has no known ortholog in nonarthropod organisms. MET has been recently confirmed as the juvenile hormone (JH) receptor playing a significant role during insect development and maturation [37]. Interestingly, in a few species of insects, like *D. melanogaster* and *Bombyx mori*, there exist the MET paralogs named germ-cell expressed (GCE) and MET2, respectively [38]. MET and GCE participate in modulating JH signaling during *D. melanogaster* development, but their functions are not fully redundant and the proteins exhibit tissue-specific distribution [39]. In turn, the MET2 protein function in *B. mori* is not yet defined [40].

## 3. Structure of bHLH–PAS Proteins

To date, our knowledge regarding the tertiary structure of bHLH–PAS proteins is limited. All determined structures comprise single isolated domains (PAS-A or PAS-B) or adjacent domains connected with flexible aa chains. C-termini, however, comprising an extensive part of proteins, have not yet been structurally characterized. These regions are not homologous to any described domains and seem to be very disordered. Consequently, it can be seen to be a huge challenge for scientists to determine their structure and combine it with specific protein functions. All bHLH–PAS structures deposited in the Protein Data Bank (PDB) are listed in Table 2 (Nuclear Resonance Magnetism (NMR) structures) and Table 3 (X-ray structures). Most of the listed assemblies correspond to heterodimers.

**Figure 2:**
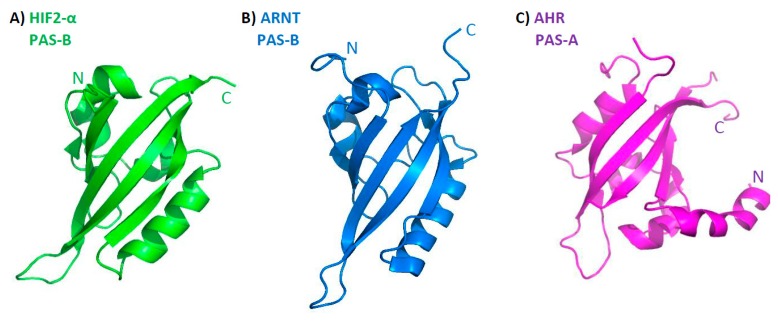
Representation of the PAS fold: a five-stranded antiparallel β-sheet is flanked by several α-helices. (**A**) HIF2-α PAS-B obtained with NMR (PDB 1P97), (**B**) ARNT PAS-B obtained with NMR (PDB 1X0O), (**C**) AHR PAS-A domain obtained with X-ray (PDB 4M4X).

The first step in determining the structure of bHLH–PAS proteins was the isolation and characterization of PAS-B domains from HIF2-α (Figure 2A) [41] and ARNT (Figure 2B) [42]. Both structures were obtained using the NMR technique and presented a fold characteristic for the PAS domain: a five-stranded antiparallel β-sheet flanked by several α-helices [42]. The next step was the crystallization of the isolated PAS-A domain of AHR (Figure 2C) and the PAS-B domains of ARNT (not shown) and HIF1-α (not shown). Interestingly, the tertiary architecture of all structurally characterized PAS domains is very conserved (Figure 2), despite the fact that their primary sequence is highly divergent (sequence identity lower than 20%) [43].

**Figure 3:**
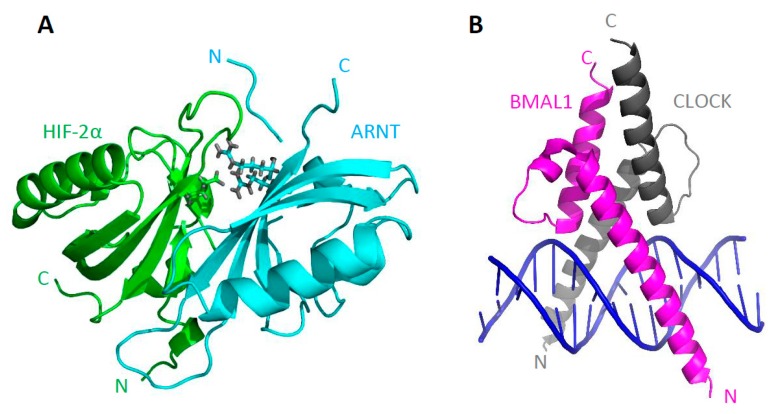
(**A**) HIF2-α PAS-B (green) and ARNT PAS-B (blue) heterodimer (3F1P, [46]). Amino acids creating a salt bridge are marked (HIF2-α E247, ARNT R362, ARNT R379). (**B**) BMAL1 bHLH (magenta) and CLOCK bHLH (grey) domains with E-box DNA (blue) (1H10, [44]).

Further experiments led to the cocrystallization of PAS-B domains from the HIF2-α/ARNT heterodimer, which revealed that these two domains form an interaction interface via their β-sheets in an antiparallel form (Figure 3A) [42]. Another measurement covering bHLH domains of BMAL1/CLOCK bound to the DNA defined domain structure and binding properties specifying interactions taking place (Figure 3B) [44]. A typical bHLH domain comprises two long α helices connected by a short loop. The first helix includes the basic domain and interacts with the major groove of the DNA [45]. All presented structures allowed an insight into the organization of bHLH–PAS proteins; however, the structure of the multidomain bHLH–PAS protein was still missing.

**Figure 4:**
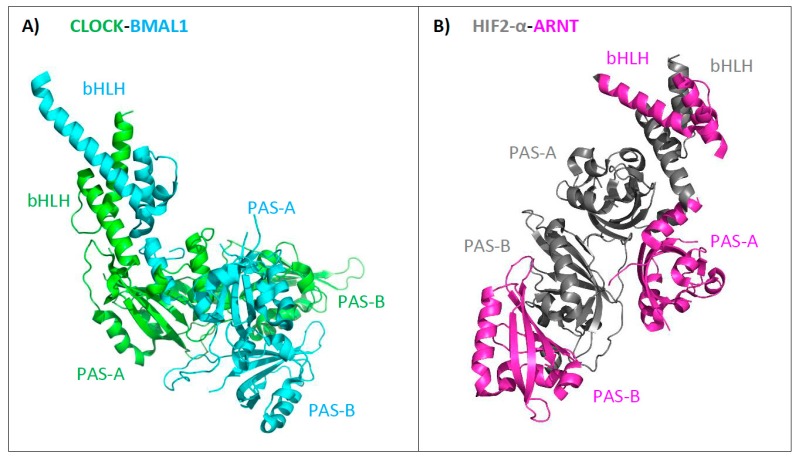
Representatives of the two groups of the bHLH–PAS heterodimers. (**A**) Overall structure of the CLOCK-BMAL1 heterodimer (4f3l, [47]), (**B**) overall structure of the HIF2-α–ARNT heterodimer (4zp4, [48]).

A turning point was the year 2012, when the first heterodimer comprising the bHLH–PAS-A/PAS-B domains (CLOCK-BMAL1) was crystallized [47] and its structure was resolved (Figure 4A). In 2015, the architecture of two other heterodimers, HIF1-α-ARNT (not shown) and HIF2-α-ARNT (Figure 4B), were obtained [48]. All determined structures present the position of the defined domains in relation to each other in the functional heterodimers. In general, the individual PAS domains are not involved in equal interactions, and the obtained structures are highly asymmetric. Importantly, two groups of heterodimers (based on BMAL-1 or ARNT proteins as a dimerization partner) present separate types of quaternary architecture. All domains in the BMAL-1 group are close spatially to each other (Figure 4A), while ARNT domains do not create intramolecular interactions and can wrap up around a partner protein (Figure 4B) [22,48].

To date, all available structural information concerns mammalian bHLH–PAS proteins. There is almost no information about the structure of proteins derived from other organisms, including invertebrate *D. melanogaster*. It would be interesting to verify evolutionary conservation of the entire bHLH–PAS fold in different organisms. The majority of reported protein structures include defined N-terminal domains, while the structural information about C-terminal regions is still missing and limited to short peptides bound to interacting proteins [22]. An example is a short motif featuring a conserved sequence LIXXL found in *D. melanogaster* MET and GCE, which represents a novel nuclear receptor (NR) box. The Docking models of the MET/GCE NR box associating peptides to the orphan nuclear receptor (FTZ-F1) ligand-binding domain (LBD) revealed their α-helical structure, necessary for hydrophobic interaction [49].

## 4. Unique Properties of the C-Terminal Domains of bHLH–PAS Proteins as IDRs

While the N-terminal part of bHLH–PAS proteins is responsible for interactions with DNA, ligands/cofactors binding, and heterodimerization, their C-termini are usually responsible for the regulation of the protein and the activity of created complexes [50]. The variability of the amino acid sequence of C-terminal fragments, their transactivation role, and the lack of homology to any described domains prompted us to ask the question about the structural character of these regions and the relationship of their character with the performed function. For a long time, it was believed that spontaneous folding into a well-defined and stable tertiary structure is required for the protein action [51]. However, it is actually known that more than 20–30% of eukaryotic proteins do not have a stable tertiary structure in physiological conditions, but at the same time still perform important biological functions. Such proteins are referred to as intrinsically disordered proteins (IDPs). Simultaneously, over 70% of proteins involved in signal transduction cascades have long intrinsically disordered regions (IDRs). Importantly, the lack of a defined structure is critical for the functionality of IDPs and IDRs [52]. Additionally, the conformational plasticity and elongated shape make them a frequent target of different kinds of post-translational modifications (phosphorylation, acetylation, methylation, and others) that regulate protein activity [53]. IDPs were identified as elements of cellular signaling which control mechanisms and protein interaction networks [54]. IDPs were also shown to take part in disease-related signaling transduction; for example, intrinsically disordered amyloid β-peptides are involved in Alzheimer’s disease [55]. Therefore, IDPs can be seen to be targets for drug design strategies.

### 4.1. In Silico Analyses of Selected bHLH–PAS Proteins

**Figure 5:**
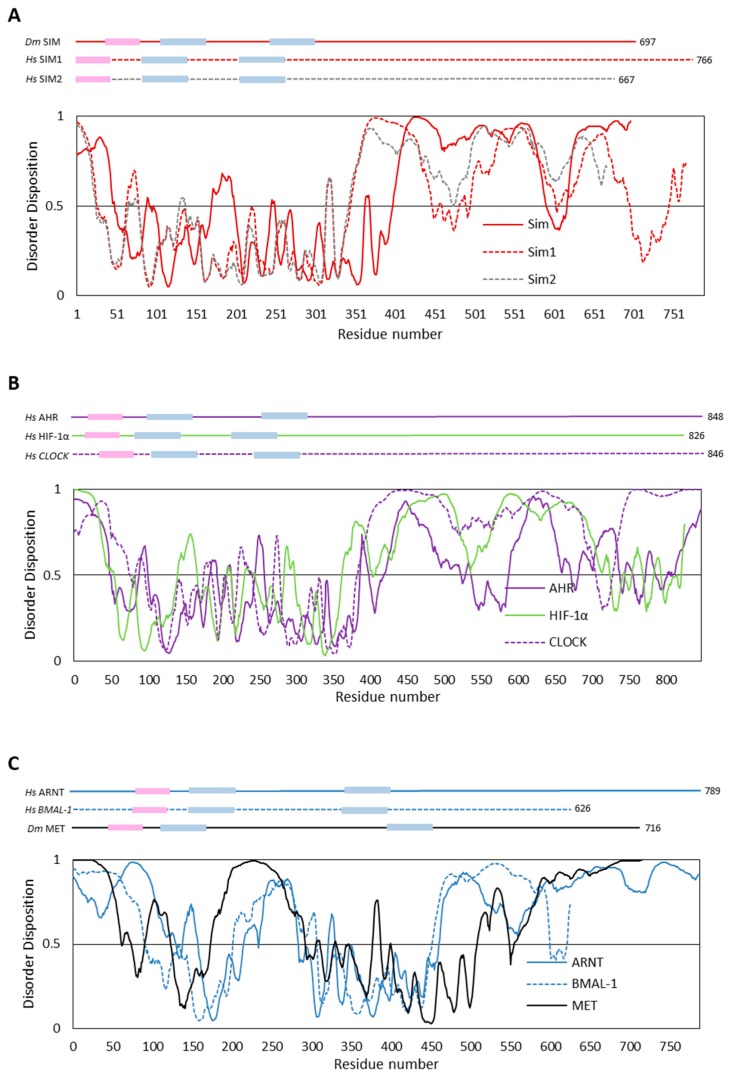
Prediction of intrinsically disordered regions. The top panel presents the domain structure of the analyzed bHLH–PAS proteins. Pink indicates the bHLH domain, whereas blue represents PAS domains. The length of the proteins is marked. The bottom panel presents a prediction of intrinsically disordered regions based on the amino acid sequence of proteins. All calculations were performed using PONDR-VLS2 software [57]. A score over 0.5 indicates disorder. (**A**) The class I proteins: *D. melanogaster* SIM (red line) and its H. sapiens orthologs SIM1 (dashed red line) and SIM2 (dashed grey line). (**B**) The class I proteins: H. sapiens AHR (violet line), HIF1-α (green line), and CLOCK (violet dashed line). (**C**) The class II proteins: H. sapiens ortholog ARNT (blue line), BMAL-1 (blue dashed line), and *D. melanogaster* Met (black line).

To estimate the occurrence of putative IDRs in bHLH–PAS proteins, we performed in silico analyses of the composition, hydropathy, and sequence complexity of amino acid sequences corresponding to selected proteins. We used the previously described human SIM1 and SIM2, as well as their *D. melanogaster* ortholog, SIM (Figure 5A), representing the class I of the family. To obtain a wider spectrum, we studied other human class I members, AHR, HIF1-α, and CLOCK (Figure 5B), which are engaged in different signal transduction pathways. As mentioned previously, class I proteins dimerize with class II proteins to form a functional complex and are crucial for heterodimer specificity. As each bHLH–PAS class II transcription factor is able to interact with different class I members, we found it to be extremely interesting to perform in silico analysis of the structure of class II members. We chose human ARNT, human BMAL1 (Figure 5C), and, additionally, *D. melanogaster* MET (Figure 5C) as a unique protein with an unknown mammalian homolog. MET can be classified as a class II bHLH–PAS family member based on its ability to not only create heterodimers with its paralog GCE, but also homodimers [56]. 

We performed in silico analysis using the predictors of intrinsically disordered regions: PONDR-VSL2 [57], PONDR-FIT [58], IUPred [59], and IsUnstruct [60]. Since the results of all the employed predictors were compatible, for the purpose of simplicity, we decided to show only one representative result (PONDR-VSL2) for each protein (Figure 5). All the results (Figure 5) substantiate our hypothesis and indicate the intrinsic character of the long C-termini. It is worth noting that for proteins representing class I (see Figure 5A,B), short ordered fragments in their C-termini are visible. Such fragments are able to act as TAD/RPD or so-called molecular recognition elements (MoREs) [61,62]. The presence of MoREs makes the interactions between partner proteins highly specific and reversible [52]. The presented results revealed some subtle differences in the regions that comprise preserved domains. The structure predicted for class I proteins (Figure 5A,B) is undeniably more ordered, while class II proteins show a marked structure relaxation in their middle part (Figure 5C), which is C-terminally linked to the PAS-A domain responsible for specificity of gene activation by bHLH–PAS proteins [63]. Such a difference explains the ability of class II proteins to serve as an interaction partner for different proteins [22]. The ability of IDRs (and IDPs) to interact with several partners is an undeniable advantage in molecular recognition processes [64]. Importantly, the resulting induced folding may differ depending on the binding partner. For example, a disordered region of p53 protein, a known cell cycle regulator and a tumor suppressor [65], folds into alpha helix or beta strand, depending on the partner protein [66].

### 4.2. The Impact of Disordered Regions on Protein Function

The flexibility and disorder detected in individual C-termini can be related to the ability of individual bHLH–PAS proteins to perform diverse functions. The differences between SIM1 and SIM2 C-termini, regarding their opposite functions (gene activation/repression), have previously been described. C-terminal regions of two other studied proteins, AHR (class I member) and ARNT (class II member), are characterized by the presence of TADs [67], in which functions are mediated by CBP/p300 and RIP140 coactivators. The C-terminal region of ARNT was additionally proposed to be a crucial activator of the estrogen receptor (ER) [68]. Interestingly, the suppression of AHR activity is also connected with the C-terminus and is mediated by the binding of the small peptide inhibitor [69]. Another repressor of the AHR signaling pathway, AHRR, is distinguished from AHR by the presence of three SUMOylation sites in its C-terminus. As shown, SUMOylation is crucial for full suppressive activity of AHRR [70].

Moreover, the C-terminus of another studied protein, HIF1-α, is characterized by the presence of TADs, and it also interacts with the CBP/p300 coactivator. The C-terminus is additionally responsible for protein stability/degradation and contains sequence motifs influencing subcellular localization: nuclear localization signal (NLS) and nuclear export signal (NES) [71,72].

Another remarkable class I bHLH–PAS protein is CLOCK, comprising a domain with histone transacetylase (HAT) activity in the C-terminus. This domain is responsible for histones acetylation, which affects the transcriptional stimulation of clock-controlled genes. Additional acetylation is performed on the R537 residue of the partner protein, BMAL1. R537 residue is located in the C-terminal part of BMAL1 and its modification facilitates the cryptochrome (CRY1)-mediated repression of specific gene transcription [73]. Importantly, CRY1 competes with the CBP/p300 coactivator for BMAL1 TAD binding, and is not able to bind the C-terminus in the paralog protein, BMAL2. Therefore, C-termini distinguish the circadian functions of these two BMAL paralogs [74].

### 4.3. Structural Analysis of bHLH–PAS C-Terminal Fragments

To date, the only structurally characterized C-terminal fragment of the bHLH–PAS protein is the *D. melanogaster* MET C-terminus (MET/C) [75]. It was shown by a series of in vitro analyses that MET/C exhibits a highly disordered character and exists in a solution in extended flexible form with predispositions for conformational changes. It is interesting to note that some short secondary motifs in the structure of MET/C have been predicted. Such short ordered fragments can be important during partner recognition and interactions. It was hypothesized that the intrinsic disorder of the C-terminal fragment was indispensable for the functionality of MET due to it modulating the protein’s action in a context-specific way. It enables cross-talk between JH signaling and other signaling pathways during *D. melanogaster* development. Previously, it was shown that Met interacts with FTZ-F1 by its C-terminus [49], thereby modulating stage-specific responses to the hormones during *D. melanogaster* metamorphosis [76]. 

As all the in vitro analyses results obtained for the MET/C [75] were consistent with the in silico studies presented above (Figure 5C), we hypothesize that the disorder character of the bHLH–PAS proteins subfamily C-terminal fragments can be a more common characteristic and also be very important for their functionality. Previously, the importance of the disordered character of regions flanking the bHLH domain of bHLH transcription factors was shown [77,78,79].

### 4.4. Structural Analysis of IDPs

While C-terminal regions of the bHLH–PAS family are considered as IDRs, it can be challenging to detect and characterize them. The reason is that IDPs and IDRs do not adopt a single stable structure and the energetically most favorable conformations can be very distinguished [80,81]. The tiny conformational changes can promote IDPs/IDRs aggregation [82]. Additionally, it was shown that IDPs/IDRs can be highly sensitive to proteolysis [83]. Currently, studies focused on the characterization of IDRs and IDPs are rapidly developing, and techniques enabling the study of proteins in solution are still improving.

There are a number of bioinformatics tools allowing primary recognition of disordered proteins. Since IDPs are characterized by the specific aa composition (a low content of hydrophobic and a high content of charged residues [84]), the Composition Profiler [85] is commonly used to compare aa distribution between the studied protein and IDPs (DisProt3.4 database)/globular proteins (PDB S25 database). Additionally, for IDPs and globular proteins distinguishing, the Uversky diagram plotting mean net charge versus mean hydrophobicity is useful [86]. Disorder predictors (like PONDR-VSL2 [57], PONDR-FIT [58], IUPred [59], and IsUnstruct [60] used in this work) allow determining the probability of IDR occurrence utilizing the neural networks, trained on selected sets of ordered and disordered sequences. Another predictor, DynaMine, provides information about protein backbone flexibility [87,88]. IDPs, once purified, can be identified by various experimental methods. First, the underestimated mobility during SDS-PAGE electrophoresis can indicate the extended and elongated shape of the protein [75]. Hydrodynamic analysis comprising Size Exclusion Chromatography (SEC) [89] and Analytical Ultracentrifugation (AUC) are commonly used to determine hydrodynamic properties, like the Stokes radius (R_S_), the sedimentation coefficients (s), and the frictional ratios (f/f_0_) [90]. The Circular Dichroism (CD) is useful for secondary structures content calculation [91]. All listed techniques allow obtaining preliminary insight into protein structure properties. 

One technique commonly used to study the overall shape and structural transitions of biological macromolecules in solution is small-angle X-ray scattering (SAXS) [92]. However, SAXS only provides limited information about the low-resolution overall shape of the molecule, so it is important to combine it with complementary high-resolution methods like NMR that present the local structure [93]. NMR offers unique opportunities that are based on analyzing the deviations from an idealized random coil devoid of any structural propensity [94]. The random coil exhibits characteristic chemical shifts, which are averages of all the possible conformations that amino acids can adopt in a solution. Therefore, NMR chemical shift deviations from random coil values can be used to evaluate the local transient secondary structure of IDPs [80]. The main problem during spectra assignments of IDPs is spectra overlapping (low chemical shifts dispersion) and a significant proton exchange with bulk water that reduces ^1^H^N^ signal intensities, which in turn leads to low signal-to-noise ratios [94]. The exchange with water can be reduced by conducting measurements in low temperature or low pH [95]. Low-resolution spectra require the development of a novel NMR technique. Recently, IDP-dedicated methods such as ^13^C-direct detected experiments, paramagnetic relaxation enhancements (PREs), or residual dipolar couplings (RDCs) have been described [96].

## 5. Conclusions

The available structure characterization of bHLH–PAS proteins is limited to the relatively well-conserved domains bHLH, PAS-A, and PAS-B. Importantly, all structures deposited in the Protein Data Bank are obtained for mammalian family members, the majority of them being heterodimers. On the other hand, the important parts of bHLH–PAS factors, which comprise their long C-termini, have not yet been structurally characterized. These fragments perform important functions in the specific modulation of protein action and for the recognition of interacting partners.

Performed in silico analysis revealed that the C-termini of representatives of the class I bHLH–PAS protein family members (SIM, SIM1, SIM2, AHR, HIF1-α, and Clock), and also class II (ARNT, BMAL1, and MET), are predicted as intrinsically disordered regions (IDRs) and are not homologous to any described domains. We discussed the known functions of the presented C-termini proteins according to their disorder character. Moreover, we proposed NMR techniques for intrinsically disordered C-termini characterization [94]. We believe that the structural properties of subsequent IDRs predicted in the sequences of bHLH–PAS transcription factors (mainly C-termini) need to be resolved for a full understanding of the way of bHLH–PAS family transcription factors function.

## Figures and Tables

**Table 1:** Mammalian class I and class II bHLH–PAS proteins [1,21,30,31,32].

Class I	Class II	Type of Signal
hypoxia-inducible factors (HIF; HIF1-α, HIF2-α, and HIF3-α)	aryl hydrocarbon receptor nuclear translocator (ARNT), also known as HIF1-β and ARNT2	regulated by hypoxia
aryl hydrocarbon receptor (AHR); aryl hydrocarbon receptor repressor (AHRR)		regulated by xenobiotics
single-minded proteins (SIM1 and SIM2)		developmentally regulated
neuronal PAS domain proteins (NPAS)		developmentally regulated
circadian locomotor output cycles protein kaput (CLOCK)	Circadian rhythm proteins (BMAL1 and BMAL2, also known as ARNTL and ARNTL2)	circadian rhythms

**Table 2:** bHLH–PAS protein structures deposited in the PDB obtained with NMR.

Form	Protein	Segment	Organism	PDB ID
monomers	HIF2-α	PAS-B domain	*Homo sapiens*	1P97
	ARNT	PAS-B domain	*Homo sapiens*	1X0O
dimer	HIF-2a:ARNT	PAS-B domains	*Homo sapiens*	2A24

**Table 3:** bHLH–PAS protein structures deposited in the PDB obtained with X-ray diffraction.

Form	Protein	Segment	Organism	PDB ID
monomers	AHR	PAS-A	*Mus musculus*	4M4X
	ARNT	PAS-B	*Homo sapiens*	2B02
	HIF1-α	PAS-B	*Homo sapiens*	4H6J
dimers	ARNT Homodimer	PAS-B	*Homo sapiens*	4EQ1
	HIF2-α:ARNT	PAS-B	*Homo sapiens*	3F1P
	HIF2-α:ARNT with artificial ligand	PAS-B	*Homo sapiens*	3F1O
	HIF2-α:ARNT	PAS-B	*Homo sapiens*	6D0C
	ARNT/HIF transcription factor/coactivator complex	PAS-B	*Homo sapiens*, *Mus musculus*	4PKY
	ARNT transcription factor/coactivator complex	PAS-B domain	*Homo sapiens, Mus musculus*	4LPZ
	HIF2-α:ARNT	bHLH; PAS-A; PAS-B	*Mus musculus*	4ZP4
	HIF2-α:ARNT with HRE DNA	bHLH; PAS-A; PAS-B	*Mus musculus*	4ZPK
	HIF1-α:ARNT with HRE DNA	bHLH; PAS-A; PAS-B	*Mus musculus*	4ZPR
	AHR:ARNT	bHLH; PAS-A	*Homo sapiens*	5NJ8
	AHR:ARNT bound to the dioxin response element (DRE)	bHLH; PAS-A	*Homo sapiens, Mus musculus*	5V0L
	AHRR:ARNT	bHLH; PAS-A; PAS-B	*Homo sapiens*, *Bos taurus*	5Y7Y
	NPAS1:ARNT	bHLH; PAS-A; PAS-B	*Mus musculus*	5SY5
	NPAS3:ARNT in complex with HRE DNA	bHLH; PAS-A; PAS-B	*Mus musculus*	5SY7
	CLOCK-BMAL1	bHLH	*Homo sapiens*	4H10
	CLOCK:BMAL1	bHLH; PAS-A; PAS-B	*Mus musculus*	4F3L

## Abbreviations

Aa	amino acid
AHR	aryl hydrocarbon receptor
AHRR	aryl hydrocarbon receptor repressor
ARNT	aryl hydrocarbon receptor nuclear translocator
bHLH–PAS	helix–loop–helix/Per-ARNT-SIM
CLOCK	circadian locomotor output cycles protein kaput
CRY1	cryptochrome
DYS	dysfusion protein
ER	estrogen receptor
GCE	germ cell-expressed protein
HAT	histone transacetylase
HIF	hypoxia-inducible factors
IDPs	intrinsically disordered proteins
IDRs	intrinsically disordered regions
JH	juvenile hormone
LBD	ligand-binding domain
MET	methoprene-tolerant protein
MoREs	molecular recognition elements
MET/C	C-terminus of the MET protein
NES	nuclear export signal
NLS	nuclear localization signal
NMR	Nuclear Resonance Magnetism
NPAS	neuronal PAS domain-containing proteins
NR	boxes nuclear receptor boxes
PAC	PAS-associated C-terminal motif
PDB	Protein Data Bank
PREs	paramagnetic relaxation enhancements
RDCs	residual dipolar couplings
SAXS	Small-angle X-ray Scattering
SIM	single-minded proteins
SIMA	similar protein
SS	spineless protein
TAD or TRD	transactivation or repression domain
TGO	TANGO protein
TRH	trachealess protein
